# Comprehensive analysis of inhibitor of differentiation/DNA-binding gene family in lung cancer using bioinformatics methods

**DOI:** 10.1042/BSR20193075

**Published:** 2020-02-11

**Authors:** Suming Xu, Yaoqin Wang, Yanhong Li, Lei Zhang, Chunfang Wang, Xueqing Wu

**Affiliations:** 1 Center of Reproductive Medicine, Children’s Hospital of Shanxi and Women Health Center of Shanxi, Xinmin North Street 13#, Taiyuan, Shanxi 030013, China; 2 Laboratory Animal Center, Shanxi Medical University, Xinjian South Road 56#, Shanxi 030001, China

**Keywords:** expression, inhibitor of differentiation/DNA-binding, lung cancer, prognostic, protein interaction network

## Abstract

The inhibitor of differentiation/DNA-binding (ID) is a member of the helix–loop–helix (HLH) transcription factor family, and plays a role in tumorigenesis, invasiveness and angiogenesis. The aims were to investigate the expression patterns and prognostic values of individual ID family members in lung cancer, and the potential functional roles. The expression levels of ID family were assessed using the Oncomine online database and GEPIA database. Furthermore, the prognostic value of ID family members was evaluated using the Kaplan–Meier plotter database. The genetic mutations of ID family members were investigated using the cBioPortal database. Moreover, enrichment analysis was performed using STRING database and Funrich software. It was found that all the ID family members were significantly down-regulated in lung cancer. Prognostic results indicated that low mRNA expression levels of ID1 or increased mRNA expression levels of ID2/3/4 were associated with improved overall survival, first progression and post progression survival. Additionally, genetic mutations of ID family members were identified in lung cancer, and it was suggested that amplification and deep deletion were the main mutation types. Furthermore, functional enrichment analysis results suggested that ID1/2/4 were significantly enriched in ‘regulation of nucleobase, nucleoside, nucleotide and nucleic acid metabolism’ for biological process, ‘transcription factor activity’ for molecular function and ‘HLH domain’ for protein domain. However, it was found that ID3 was not enriched in the above functions. The aberrant expression of ID family members may affect the occurrence and prognosis of lung cancer, and may be related to cell metabolism and transcriptional regulation.

## Introduction

Lung cancer is one of the most common types of malignancies worldwide, and has higher morbidity and mortality rates compare with other malignant tumors [1]. As a threat to human health, lung cancer has been the focus of the health research field worldwide [2]. Currently, the survival of patients with lung cancer relies on quick and accurate clinical diagnosis. However, most patients have intermediate or advanced lung cancer at the time of diagnosis, and thus have a poor survival rate [3]. Therefore, it is important to investigate potential biomarkers that are related to the occurrence and prognosis of lung cancer, and to identify the possible molecular mechanisms.

The inhibitor of differentiation/DNA-binding (ID) belongs to helix–loop–helix (HLH) family of transcription factors. ID can bind to HLH transcription factors to inhibit their binding to DNA, resulting in the inhibition of cell differentiation and promotion of cell proliferation [4]. In humans, the ID family members consist of four members: *ID1, ID2, ID3* and *ID4*. Previous studies have demonstrated that ID proteins play important roles in proliferation, apoptosis, differentiation, invasion, metastasis and angiogenesis in various human tumor types [5–7]. However, the role of ID family members in lung cancer is not fully understood.

Kamalian et al. [8] found that the expression levels of *ID1, ID2* and *ID3* were increased in human small cell lung cancer (SCLC) tissues and cell lines, and that only *ID4* was increased in SCLC tissues. In addition, Kamalian et al. [8] revealed that the increased expression level of *ID2* in the cytoplasm of patients with SCLC is significantly associated with increased survival. Therefore, *ID2* may be used as a prognostic factor of patients with SCLC. Cheng et al. also reported that the expression level of *ID1* is increased in lung cancer cell lines and tissues, and promotes lung cancer cell proliferation and tumor growth via the Akt-related signaling pathway [9]. Furthermore, elevated expressions levels of *ID1* and *ID3* are associated with SCLC tumorigenicity by enhancing angiogenesis and suppressing apoptosis [10]. Moreover, Li et al. found that the effects of *ID1* in non-SCLC (NSCLC) cells may promote proliferation, migration and invasion by activating the NF-κB signaling pathway [11]. In addition, Qi et al. found that *ID4* could inhibit cisplatin-induced apoptosis via the p38 MAPK pathway [12]. In NSCLC, which affects 80% of patients with lung malignancies, *ID2* may be involved in dedifferentiation, and could be used as a prognosis marker for patients with poorly differentiated tumors [13]. Therefore, ID family members may provide new targets and biomarkers for the treatment and prognosis of lung cancer.

Bioinformatics analysis has been widely applied in the biology research field. The present study investigated the role of individual ID family members in lung cancer using large databases, which included the Oncomine database, Gene Expression Profiling Interactive Analysis (GEPIA) database, Kaplan–Meier plotter, cBio Cancer Genomics Portal (cBioPortal) database, Search Tool for the Retrieval of Interacting Genes (STRING) database and Funrich software. The aims of the present study were to examine the characteristics of individual ID family members in lung cancer, including the expression levels, prognostic values and potential functions, to further the understanding of ID proteins.

## Materials and methods

### Oncomine analysis

The Oncomine online database (www.oncomine.org) is an online cancer microarray database, which is used to compare the expression between tumors and normal tissues in various cancer types [14]. The selection criteria included: *P* < 0.05, 2-fold change and gene rank in the top 10%.

### GEPIA database

The GEPIA database (http://gepia.cancer-pku.cn/) is a newly developed interactive web server for comparing the gene expression profile in cancer and paired normal tissues [15]. The present study compared the expression profile of ID family members in lung cancer using the GEPIA database.

### Kaplan–Meier analysis

The prognostic values of ID family members in lung cancer were assessed using the Kaplan–Meier plotter (www.kmplot.com), which can evaluate the effect of 54,000 genes on survival rate in 21 cancer types including breast cancer, lung cancer and ovarian cancer [16,17]. A total of 1926 lung cancer samples were used to assess first progression (FP), overall survival (OS) and post progression survival (PPS), using a Kaplan–Meier survival plot. In addition, the present study evaluated the associations of the ID family members with different clinical parameters for OS, including the histology, clinical stages, pathological grades, American Joint Committee on Cancer (AJCC) stages, sex and smoking status. The log-rank *P* < 0.05 was considered to indicate a statistically significant difference.

### cBioPortal database

The cBioPortal database (http://cbioportal.org) is an open-access resource for the interactive investigation of multidimensional cancer genomics datasets [18]. The genetic mutations of ID family members in different cancer types were obtained, according to the online cBioPortal database.

### String database

The STRING database (https://string-db.org/) is a search tool for the retrieval of interacting genes, which is used to assess protein–protein interaction (PPI) information [19]. The gene networks of ID family member genes were constructed, and the interactions with a combined score >0.4 were selected.

### Funrich analysis

Functional enrichment analysis of the interacting proteins was performed using Funrich software (version 3.1.3), which is an open-access standalone functional enrichment and interaction network analysis tool [20]. Moreover, the functional analysis of related gene interactions with ID family members was performed using Funrich software.

## Results

### Transcription expression levels of ID family members in human cancer types

**Figure 1:**
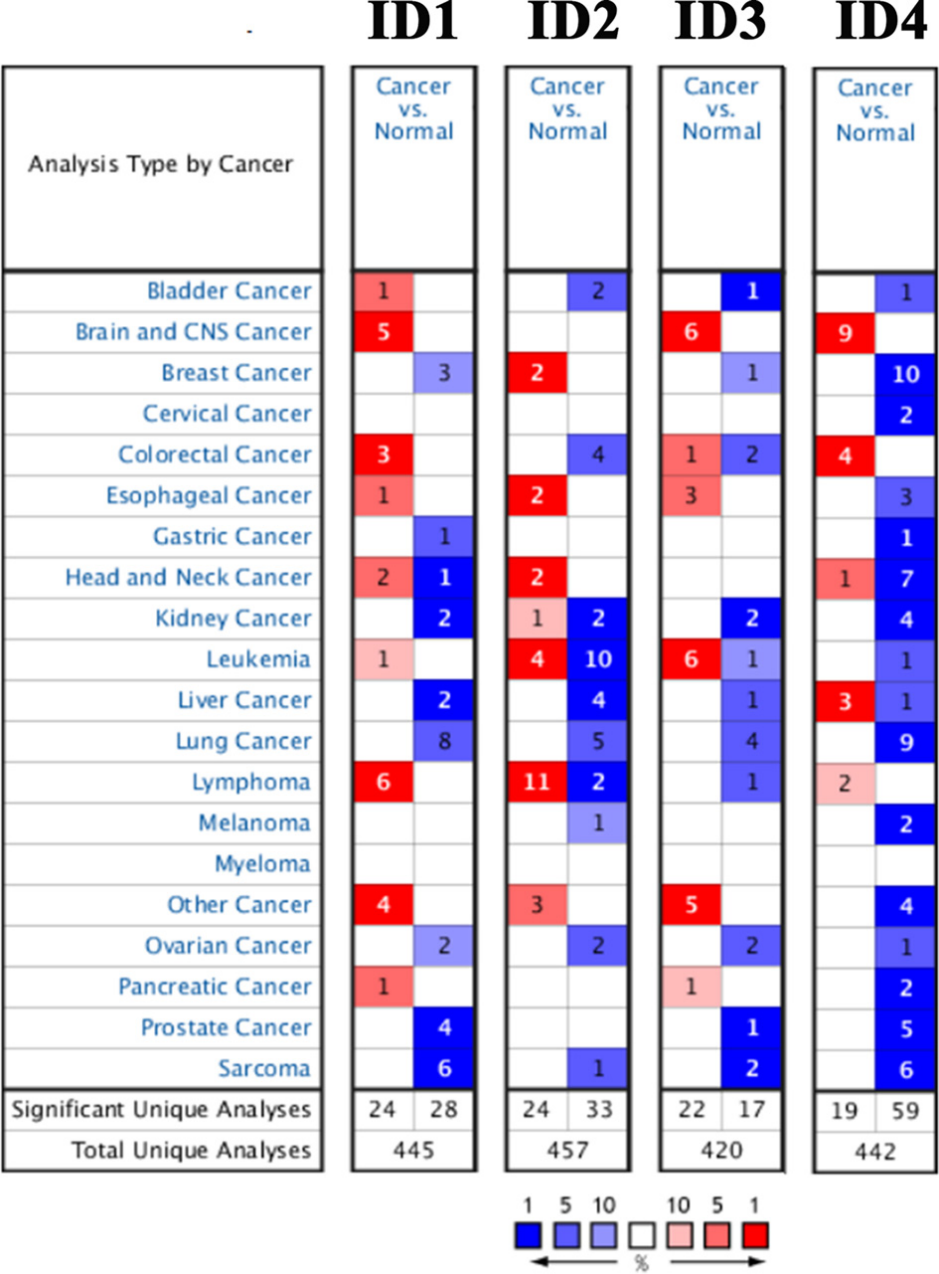
mRNA expression levels of ID family genes in the Oncomine database The graphic demonstrates the number of datasets with statistical significance. Red, up-regulation; blue, down-regulation. The number in each cell indicates the datasets that met the set threshold in each cancer type. Cell color was defined as the gene rank percentile for analyses within the cell. ID, inhibitor of differentiation/DNA-binding.

The present study used the Oncomine online databases to compare the transcription expression levels of ID family members between human cancer and normal tissues. As shown in Figure 1, the database containing the genes of *ID1, ID2, ID3* and *ID4* had a total of 445, 457, 420 and 442 unique studies, respectively. It was found that all ID family members were significantly down-regulated in lung cancer.

Furthermore, the present study investigated the gene expression levels of ID family members in different lung cancer datasets (Table 1). In the Bhattacharjee dataset [21], *ID1* was significantly decreased in different lung cancer types compared with normal samples; lung adenocarcinoma had a fold change of -11.038, small cell lung carcinoma had a fold change of -11.150 and lung carcinoma tumor had a fold change of -45.049. Similar results were identified in lung adenocarcinoma in the Beer dataset [22], Su dataset [23], Landi dataset [24], Selamat dataset [25] and Okayama dataset [26]. For *ID2*, a decreased expression level was found in lung adenocarcinoma compared with normal samples in the Selamat [25], Beer [22], Bhattacharjee [21] and Su [23] datasets. Furthermore, in the Hou dataset [27] it was demonstrated that the expression level of *ID2* was decreased in squamous cell lung carcinoma, with a fold change of -2.485. Moreover, a low expression level of *ID3* was found in lung adenocarcinoma in the Selamat dataset [25], Landi dataset [24], Su dataset [23] and Okayama dataset [26]. Furthermore, *ID4* was significantly down-regulated in lung adenocarcinoma in the Okayama [26], Beer [22], Landi [24], Su [23], Garber [28], Stearman [29] and Hou [27] datasets, and also in squamous cell lung carcinoma (Garber [28] and Hou [27] datasets).

**Table 1:** The significant change of IDs expression in different types of lung cancer (Oncomine database)

	Dataset	Types of lung cancer versus lung	Sample	Fold change	*P*-value	*t*-test
*ID1*	Bhattacharjee	Lung adenocarcinoma versus normal	149	-11.038	5.25E-7	-6.259
		Small cell lung carcinoma versus normal	23	-11.150	1.25E-5	-6.055
		Lung carcinoid tumor versus normal	37	-45.049	2.28E-9	-7.724
	Beer	Lung adenocarcinoma versus normal	96	-5.242	1.10E-8	-6.132
	Su	Lung adenocarcinoma versus normal	66	-3.186	1.51E-7	-6.099
	Landi	Lung adenocarcinoma versus normal	107	-2.364	4.98E-12	-7.718
	Selamat	Lung adenocarcinoma versus normal	116	-3.080	9.60E-16	-9.481
	Okayama	Lung adenocarcinoma versus normal	246	-2.652	1.57E-9	-8.345
*ID2*	Selamat	Lung adenocarcinoma versus normal	116	-3.864	5.22E-27	-14.089
	Beer	Lung adenocarcinoma versus normal	96	-2.231	3.90E-7	-6.616
	Bhattacharjee	Lung adenocarcinoma versus normal	203	-4.723	2.09E-5	-5.185
	Su	Lung adenocarcinoma versus normal	66	-2.050	2.14E-7	-5.852
	Hou	Squamous cell lung carcinoma versus normal	156	-2.485	2.38E-11	-9.385
*ID3*	Selamat	Lung adenocarcinoma versus normal	116	-4.415	1.21E-27	-14.460
	Landi	Lung adenocarcinoma versus normal	107	-2.116	3.96E-16	-9.520
	Su	Lung adenocarcinoma versus normal	66	-3.906	1.50E-6	-5.575
	Okayama	Lung adenocarcinoma versus normal	246	-2.638	1.62E-11	-10.739
*ID4*	Okayama	Lung adenocarcinoma versus normal	246	-4.115	1.48E-28	-17.724
	Beer	Lung adenocarcinoma versus normal	96	-4.988	7.24E-10	-7.656
	Landi	Lung adenocarcinoma versus normal	107	-3.754	6.51E-25	-13.467
	Su	Lung Adenocarcinoma versus Normal	66	-3.259	5.44E-11	-8.206
	Garber	Lung Adenocarcinoma versus Normal	45	-2.495	4.65E-6	-6.296
		Squamous Cell Lung Carcinoma versus Normal	19	-3.193	7.94E-5	-4.885
	Stearman	Lung adenocarcinoma versus normal	39	-2.478	1.50E-7	-6.829
	Hou	Lung adenocarcinoma versus normal	110	-5.899	1.42E-15	-10.443
		Squamous cell lung carcinoma versus normal	92	-7.876	3.49E-18	-14.320

### Expression levels of ID family members in lung cancer

**Figure 2:**
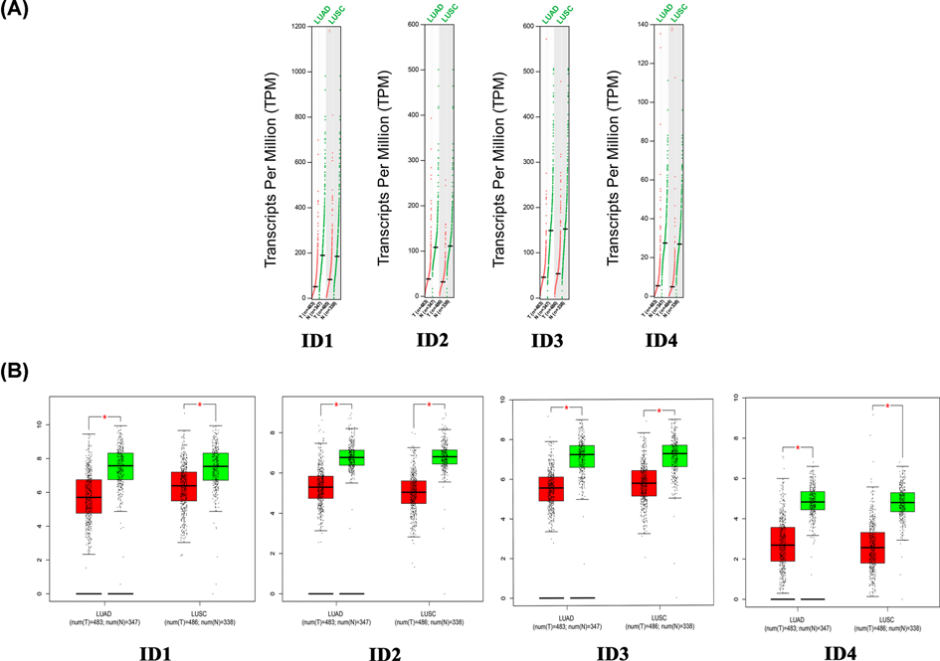
Expression of ID family members in lung cancer in the GEPIA database (**A**) Expression profile of ID family members in lung cancer. Red trace, tumor samples; green trace, normal samples. (**B**) Boxplot results of the expression levels of ID family members in lung cancer. A *t*-test was used to compare the expression level differences between tumor and normal tissues (*P* < 0.01). *Y*-axis represents log_2_(TPM+1). Red box, tumor samples; green box, normal samples. T, tumor; N, normal; LUAD, lung adenocarcinoma; LUSC, lung squamous cell carcinoma; ID, inhibitor of differentiation/DNA-binding.

**Figure 3:**
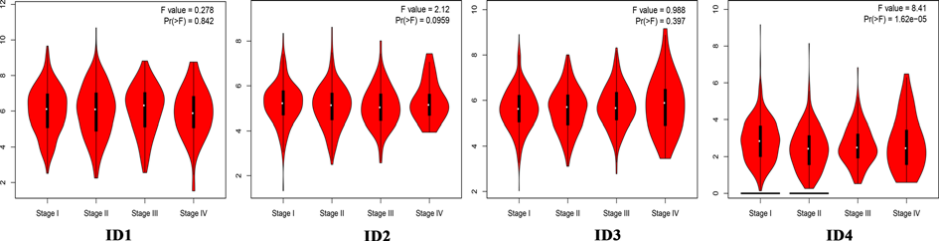
Expression of the ID family members in lung cancer stages The *Y*-axis represents log_2_(TPM+1). ID, inhibitor of differentiation/DNA-binding.

Using the GEPIA database, the present study compared the mRNA expression level of individual ID family members between lung cancer tissues and normal lung tissues. The present results demonstrated that the expression levels of ID family member genes were significantly decreased in lung adenocarcinoma and lung squamous cell carcinoma tissues compared with normal tissues (Figure 2). Furthermore, the present study analyzed the expression levels of ID family members in different lung cancer stages. It was found that *ID4* had significantly different expression levels in the various tumor stages, whereas there was no obvious difference in expression levels of *ID1, ID2* and *ID3* (Figure 3).

### Prognosis analysis of ID family members in lung cancer

**Figure 4:**
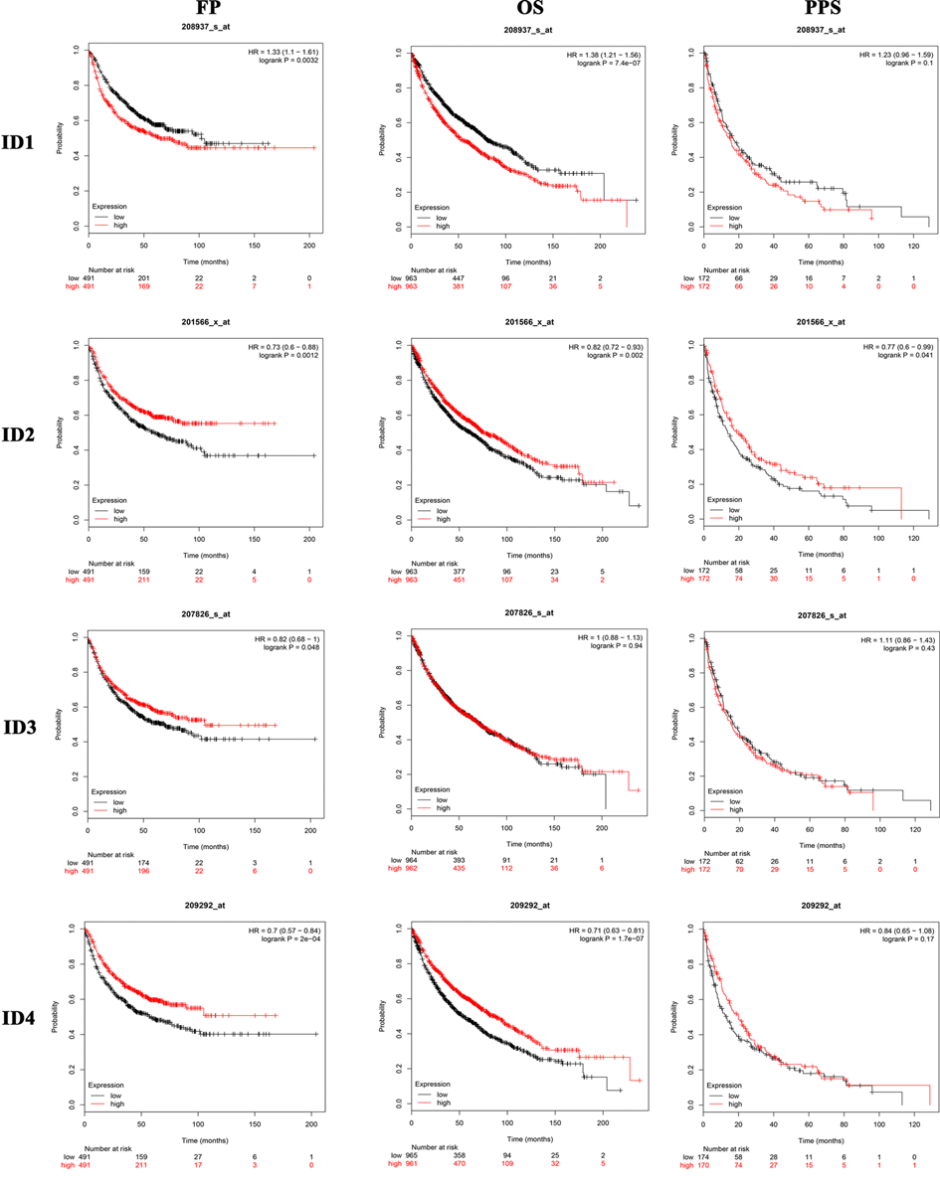
Relationship between the expression level of individual ID family members and the prognosis of patients with lung cancer ID, inhibitor of differentiation/DNA-binding; OS, overall survival; FP, progression-free survival; PPS, post-progression survival.

The present study systematically performed Kaplan–Meier survival analysis according to the mRNA expression of individual ID family members in lung cancer (http://www.kmplot.com/analysis/index.php?p=service&cancer=lung). It was found that the mRNA expression levels of *ID1/2/3/4* had a significant effect on OS, FP and PPS in patients with lung cancer (*P* < 0.05; Figure 4). Therefore, patients with lung cancer with a low mRNA expression level of *ID1* or high mRNA expression levels of *ID2/3/4* were predicted to have longer OS, FP and PPS.

Furthermore, the present study investigates the association of the expression levels of individual ID family members with various clinical parameters of lung cancer, including histology, clinical stages, pathological grades, AJCC stages, sex and smoking status (Table 2). For histology, *ID1* mRNA expression level was significantly associated with unfavorable OS in adenocarcinoma and squamous cell carcinoma. However, *ID2* and *ID4* mRNA expression levels showed a favorable association with OS in adenocarcinoma. For clinical stages, *ID1* mRNA expression level was associated with unfavorable OS in patients with stage 1 lung cancer. On the contrary, mRNA expression levels of *ID2* and *ID4* were related with favorable OS. However, it was found that ID family member mRNA expression levels showed no correlation with stages 2 and 3 of lung cancer. For pathological grades, only *ID1* was identified to be associated with unfavorable OS in patients with stage 2 lung cancer. For AJCC stages, it was demonstrated that *ID1* (AJCC stage N0), *ID2* (AJCC stage M0) and *ID3* (AJCC stage T4) were significantly associated with unfavorable OS in lung cancer. However, *ID4* (AJCC stage T1) was associated with favorable OS in lung cancer. Moreover, *ID1* was significantly correlated with poor OS in female patients with lung cancer, while *ID2/3/4* were significantly correlated with improved OS in female patients with lung cancer. In addition, it was found that *ID1* was significantly correlated with poor OS in male patients with lung cancer. The present results suggested that *ID1* mRNA expression level was significantly associated with poor OS in smokers and non-smokers. However, *ID2* and *ID4* mRNA expression levels were associated with improved OS in patients with lung cancer without a smoking history.

**Table 2:** Relation of the expression of individual ID family members with OS in lung cancer patients with different clinical parameters

Subtypes	ID1		ID2		ID3		ID4	
	HR (95% CI)	Log rank P	HR (95% CI)	Log rank P	HR (95% CI)	Log rank P	HR (95% CI)	Log rank P
Histology								
Adenocarcinoma	1.82(1.43–2.32)	1.0e-6	0.56(0.44–0.71)	1.1e-6	1.15(0.91–1.45)	0.25	0.46(0.36–0.58)	1.0e-10
Squamous cell carcinoma	1.4(1.11–1.78)	0.005	1(0.79–1.26)	0.98	0.9(0.71–1.15)	0.41	0.9(0.71–1.14)	0.4
Stage								
1	2.38(1.78–3.18)	1.5e-9	0.5(0.38–0.67)	8.3e-7	0.98(0.75–1.29)	0.89	0.4(0.3–0.53)	3.7e-11
2	1.27(0.87–1.83)	0.21	0.91(0.63–1.32)	0.63	0.9(0.63–1.3)	0.58	0.79(0.55–1.14)	0.21
3	1.19(0.69–2.06)	0.54	1.15(0.67–2)	0.61	1.54(0.89–2.67)	0.12	1.3(0.75–2.24)	0.35
4	NA	NA	NA	NA	NA	NA	NA	NA
Grade								
I	1.4(0.98–2.01)	0.065	0.95(0.66–1.36)	0.78	0.87(0.61–1.25)	0.46	1.06(0.74–1.52)	0.75
II	1.46(1.07–2)	0.017	1.25(0.91–1.72)	0.16	0.93(0.68–1.27)	0.65	0.94(0.69–1.29)	0.72
III	1.21(0.63–2.33)	0.57	1.02(0.53–1.96)	0.95	0.64(0.33–1.25)	0.19	1.01(0.53–1.95)	0.97
AJCC stage T								
1	1.23(0.92–1.63)	0.16	0.92(0.69–1.22)	0.54	0.88(0.67–1.17)	0.39	0.67(0.5–0.89)	0.0059
2	1.18(0.95–1.47)	0.14	1.07(0.86–1.33)	0.56	0.95(0.76–1.18)	0.62	1.07(0.86–1.34)	0.53
3	1.26(0.77–2.06)	0.35	1.07(0.65–1.77)	0.79	0.85(0.51–1.4)	0.51	0.91(0.56–1.49)	0.71
4	1.22(0.65–2.29)	0.53	0.91(0.48–1.71)	0.76	2.48(1.27–4.83)	0.006	1.16(0.62–2.18)	0.65
AJCC stage N								
0	1.31(1.06–1.61)	0.013	0.98(0.79–1.2)	0.81	0.96(0.78–1.19)	0.71	0.91(0.74–1.12)	0.36
1	1.2(0.88–1.65)	0.24	1.21(0.8–1.65)	0.24	0.88(0.64–1.2)	0.41	0.85(0.62–1.16)	0.3
2	1.32(0.88–1.98)	0.18	0.94(0.63–1.42)	0.78	1.45(0.96–2.2)	0.077	0.9(0.6–1.35)	0.61
AJCC stage M								
0	1.22(0.99–1.5)	0.062	1.24(1.01–1.53)	0.04	0.94(0.77–1.16)	0.57	0.84(0.68–1.04)	0.1
1	NA	NA	NA	NA	NA	NA	NA	NA
Gender								
Female	1.27(1–1.6)	0.047	0.74(0.58–0.93)	0.01	0.73(0.57–0.92)	0.0068	0.59(0.47–0.75)	1.2e-5
Male	1.45(1.24–1.7)	4.3e-6	0.85(0.73–1)	0.05	1.17(1–1.37)	0.053	0.8(0.69–0.94)	0.0061
Smoking history								
Exclude those never smoked	1.55(1.26–1.92)	3.2e-5	0.87(0.71–1.07)	0.19	0.97(0.79–1.19)	0.79	0.92(0.74–1.13)	0.41
Only those never smoked	1.81(1.03–3.19)	0.038	0.32(0.17–0.59)	0.00011	0.74(0.42–1.29)	0.29	0.36(0.19–0.67)	0.00078

### Mutation of ID family members in lung cancer

**Figure 5:**
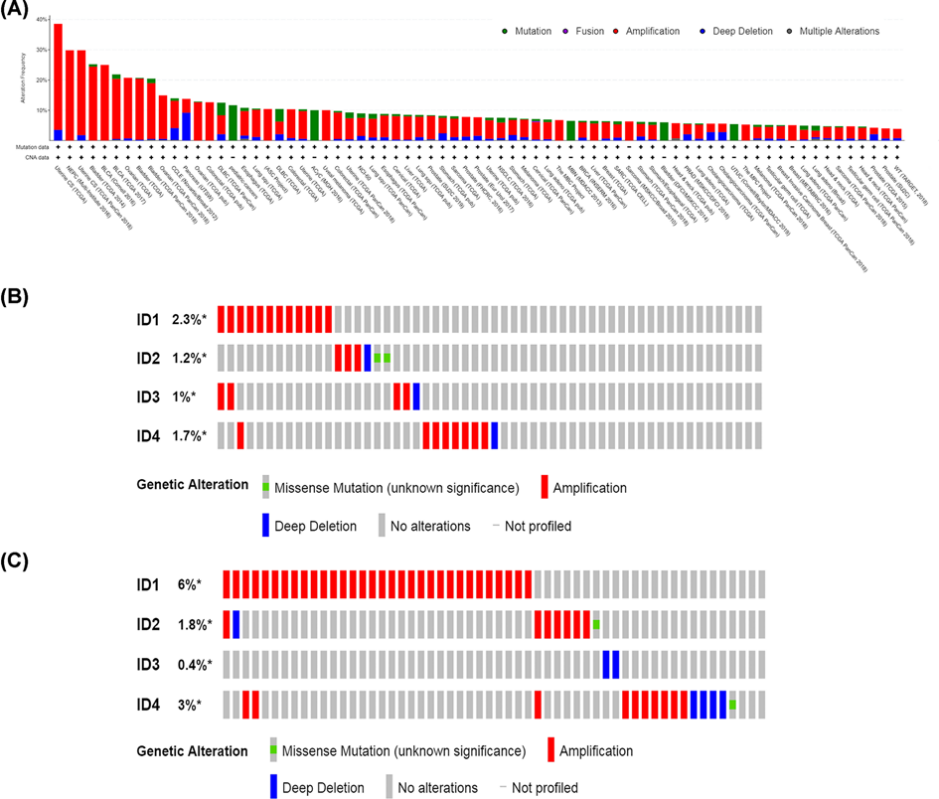
Analysis of ID family genetic mutations using the cBioPortal database (**A**) Genetic mutation frequencies of ID family members in various carcinoma types. Green, genetic mutations; purple, gene fusions; red, gene amplifications; blue, deep deletions; gray, multiple alterations. (**B**) ID family genetic mutation frequencies and mutation types in 584 patients with lung adenocarcinoma. Red, gene amplifications; blue, deep deletions; green, missense mutations. (**C**) ID family genetic mutation frequencies and mutation types in 511 patients with lung squamous cell carcinoma. Red, gene amplifications; blue, deep deletions; green, missense mutations. ID, inhibitor of differentiation/DNA-binding.

Genetic mutations in ID family members in different cancer types were assessed using cBioPortal. The ID family member genetic mutations were determined in 256 cancer studies, which included 77,879 samples. As shown in Figure 5A, genetic mutations in ID family members were present in different lung cancer types, including lung squamous cell carcinoma and lung adenocarcinoma, compared with other cancer types. Furthermore, ID family genetic mutation frequencies and types in lung adenocarcinoma (TCGA; Provisional; 586 total samples) and lung squamous cell carcinoma (TCGA; Provisional; 511 total samples) are shown in Figure 5B and C. In lung adenocarcinoma (TCGA; Provisional), the mutation frequencies of *ID1/2/3/4* were 2.3, 1.2, 1 and 1.7%, respectively, and gene amplification accounted for the majority of genetic modifications. In lung squamous cell carcinoma (TCGA; Provisional), the mutation frequencies of *ID1/2/3/4* were 6, 1.8, 0.4 and 3%, respectively, and gene amplification accounted for the majority of genetic changes for *ID1/2/4*, while deep deletions accounted for the majority of changes for *ID3*.

### Functional enrichment analysis of ID family members

**Figure 6:**
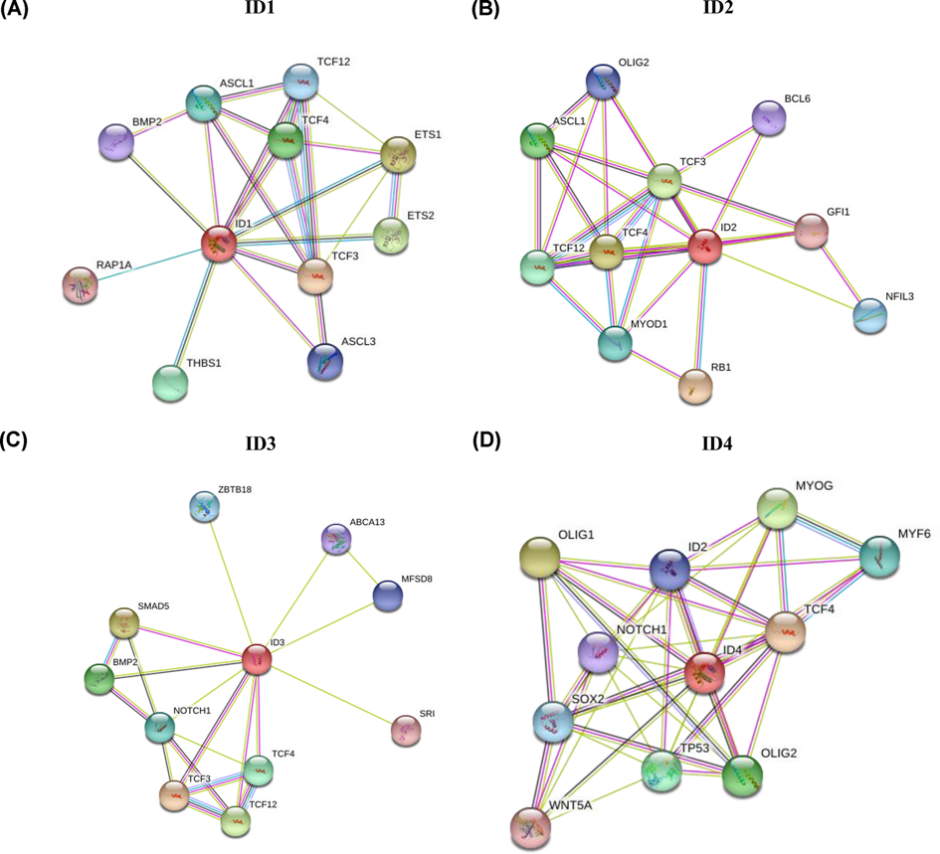
Protein–protein interaction network of individual ID family members from the STRING database (**A**) Interaction network of *ID1*. (**B**) Interaction network of *ID2*. (**C**) Interaction network of *ID3*. (**D**) Interaction network of *ID4*. ID, inhibitor of differentiation/DNA-binding.

**Figure 7:**
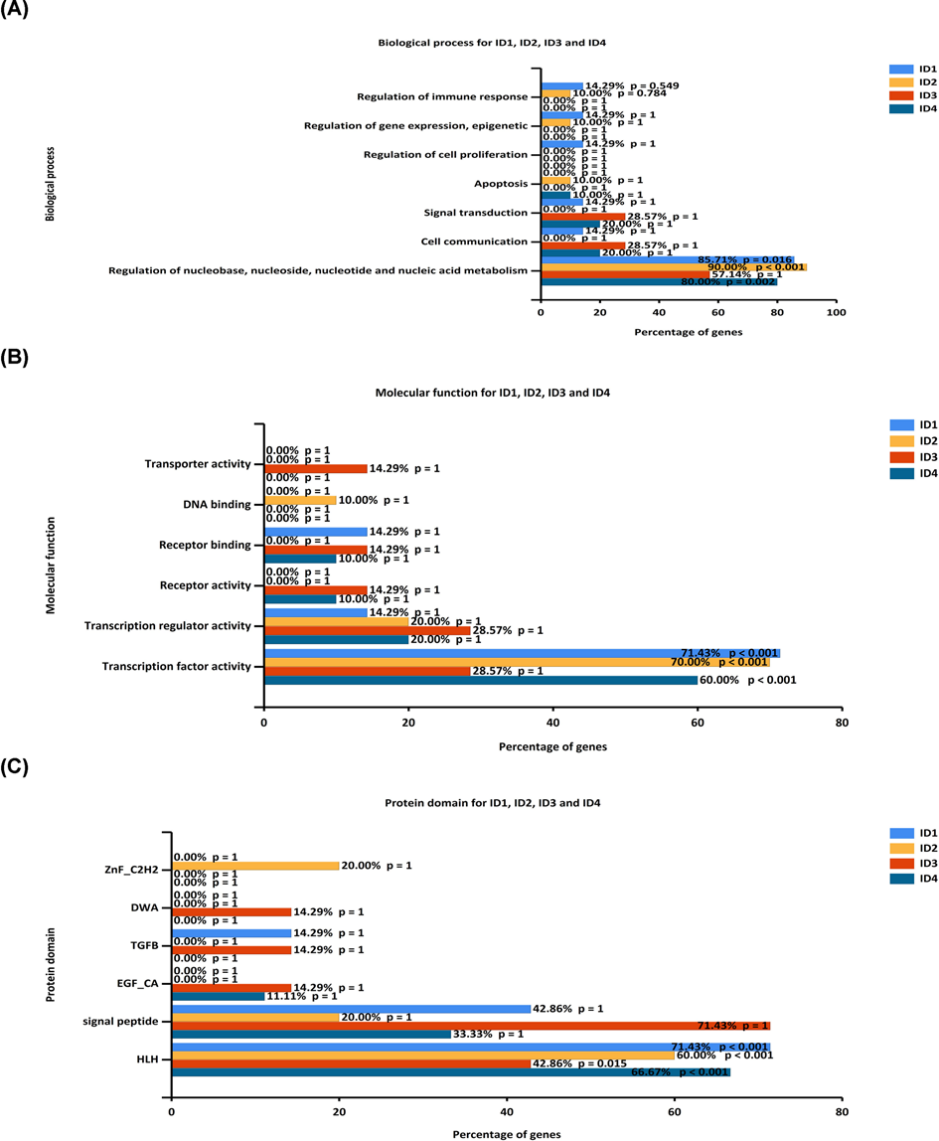
Functional enrichment analysis of potential targeted genes (**A**) Potential biological processes of ID family genes were identified using FunRich. (**B**) Potential molecular functions of ID family genes were identified using FunRich. (**C**) Potential protein domains of ID family genes were identified using FunRich.

To investigate the interactions of the ID family genes, the present study constructed PPI networks using STRING data (Figure 6). Furthermore, functional enrichment analysis of potential target genes was performed using Funrich software (Figure 7). For biological process, *ID1/2/4* were significantly enriched in ‘Regulation of nucleobase, nucleoside, nucleotide and nucleic acid metabolis’ (85.71, 90.00 and 80.00%; P<0.05). For molecular function, *ID1/2/4* were significantly enriched in ‘Transcription factor activity’ (71.43, 70.00 and 60.00%; *P* < 0.05). It was found that for protein domain, *ID1/2/4* were significantly enriched in ‘HLH domain’ (71.43, 60.00 and 66.67%; *P* < 0.05). However, *ID3* was not significantly enriched in the above aspects.

## Discussion

The development of molecular biology technology and bioinformatics has helped to understand the molecular biological characteristics of ID family members and their role in tumorigenesis. However, to the best of our knowledge, there are few studies on the role and significance of ID family members in lung cancer. Therefore, the present study investigated the expression patterns, prognostic values and potential functions of ID family members in lung cancer using bioinformatics methods. Thus, the present results may facilitate the development of future studies to identify potential therapeutic targets in lung cancer.

The present study analyzed the expression levels of ID family members in human tumors using the Oncomine database. The present results suggested that all ID family members had low expression levels in lung cancer. Furthermore, the experimental results based on the GEPIA database showed that the expression levels of ID family members were significantly decreased in lung cancer tissues, including lung adenocarcinoma and lung squamous cell carcinoma. In addition, it was also found that the expression level of *ID4* was significantly different in various tumor stages. Therefore, the present results suggested that the expression levels of ID family members may be linked to the pathogenesis of lung cancer. In relation to this, Zhou et al. reported that the mRNA expression levels of *ID1, ID3* and *ID4* were significantly lower in breast cancer tissues compared with normal tissues [30]. However, the present results are inconsistent with other previous results [8–10], and these differences may be due to the small sample size and discrepancies in detection methods amongst the different studies.

In order to understand the relationship between ID family members and lung cancer, the present study performed a prognostic analysis using the Kaplan–Meier Plotter. It was demonstrated that increased *ID2/3/4* mRNA expression levels were associated with improved OS, FP and PPS. However, decreased *ID1* mRNA expression level was associated with improved OS, FP and PPS. Therefore, the present results suggested that low mRNA expression level of *ID1* or high mRNA expression levels of *ID2/3/4* predicted an improved survival in patients with lung cancer. In addition, the present study assessed the associations of the ID mRNA expression levels with distinct clinical parameters for OS, including histology, clinical stages, pathological grades, AJCC stages, sex and smoking status. For histology, a low mRNA expression level of *ID1* was significantly associated with favorable OS in the following: Adenocarcinoma, squamous cell carcinoma, patients with stage 1 lung cancer, Grade II, AJCC stage N0, sex and smoking history. However, low mRNA expression levels of *ID2* showed an unfavorable OS in the following: Adenocarcinoma, patients with stage 1 lung cancer, female patients and patients without a smoking history. Moreover, a low mRNA expression level of *ID4* was found to be associated with unfavorable OS in the following categories: Adenocarcinoma, patients with stage 1 lung cancer, sex and patients without a smoking history. It was also demonstrated that *ID3* mRNA expression level was significantly associated with unfavorable OS in AJCC stage T4, while *ID3* showed an association with a favorable OS in female patients. Thus, the present results suggested that *ID1/2/4* mRNA expression levels were correlated with pathological type, sex and smoking history in patients with lung cancer.

Currently, it is not fully understood whether IDs act as an oncogenes or a tumor suppressor genes in different tumor types due to a lack of identification of genetic alternations in ID genes. Li et al. found that 26.7% of *ID3*^−/−^ mice developed lymphoma, while none of the *ID3*^+/+^ or *ID3*^+/−^ mice had lymphoma. Therefore, a deficiency of the *ID3* gene increases the possibility of γδ T-cell lymphoma [31]. However, to the best of our knowledge, there was no previous studies investigating genetic alterations of ID genes in other cancer types. Thus, the present study evaluated the mutations of ID family members in patients with lung cancer using the cBioPortal database. The present results suggested that there were mutations in *ID1/2/3/4*, which were predominately amplification and deep deletion mutations.

Lung cancer not only is caused by the differential expression of ID family members, but also may be caused by the interaction between related genes [32]. Therefore, the present study constructed PPI networks using STRING data for individual ID family members. Furthermore, the possible functions of ID related genes were investigated using Funrich software, including biological process, molecular function and protein domain. It was found that *ID1/2/4* were significantly enriched in ‘Regulation of nucleobase, nucleoside, nucleotide and nucleic acid metabolis’, ‘Transcription factor activity’ and ‘HLH domain’. However, *ID3* was not significantly enriched in the above factors. Therefore, the present results indicated that ID family members may be involved in cell metabolism and transcription regulation.

Transcription factors are a group of sequence-specific binding proteins that can activate or inhibit transcription via a transactivation or transrepression domains [33]. Previous studies have indicated that transcription factors are involved in regulating cell differentiation, proliferation and apoptosis, and play significant roles in the occurrence and development of tumors [34]. In the human genome, the C2H2zinc­finger domain, homeodomain and HLH domain are the main types of transcription factor, and accounted for >80% of human genome [35]. Therefore, it is important to study the role of transcription factors in lung cancer. A nucleotide is an essential nutrient required to maintain the rapid proliferation of tumor cells, and nucleotide synthesis is regulated by many enzymes, genes and various metabolic pathways [36,37]. Moreover, previous studies have reported that inactivation of tumor suppressors and activation of oncogenes can promote the occurrence and development of tumors by regulating the biosynthesis of nucleotides [38]. Therefore, the mechanism of cell metabolism and transcriptional regulation of ID family genes, and related genes in the pathogenesis of lung cancer may be an important focus for future research. In addition, the present study supports the initiation of future studies to investigate the mechanism of tumor occurrence and development, to facilitate the development of prevention and treatment strategies.

In conclusion, the present study investigated the role of ID family members in lung cancer, in relation to mRNA expression levels, prognostic values, genetic mutations and functional enrichment analysis. The present results suggested that genetic mutations and mRNA expression levels were abnormal in patients with lung cancer. Furthermore, decreased *ID1* or increased *ID2/3/4* expression levels predicted an improved survival, and it was found that these proteins were involved in cell metabolism and transcription regulation. Therefore, ID family members may be used as biomarkers for the occurrence and prognosis of lung cancer. However, further study is required to assess the expression levels and molecular mechanisms of ID family members.

## Abbreviations

cBioPortal: cBio Cancer Genomics Portal
FP: progression-free survival
GEPIA: Gene Expression Profiling Interactive Analysis
HLH: helix–loop–helix
ID: inhibitor of differentiation/DNA-binding
NSCLC: non-small cell lung cancer
OS: overall survival
PPI: protein–protein interaction
PPS: post-progression survival
SCLC: small cell lung cancer
STRING: Search Tool for the retrieval of interacting Genes
